# Beyond pulmonary arterial hypertension: sotatercept across the advanced heart failure and transplant continuum

**DOI:** 10.3389/frtra.2026.1966659

**Published:** 2026-09-29

**Authors:** Andrew Xanthopoulos, Christos Kourek, Dimitrios E. Magouliotis, Filippos Triposkiadis, Alexandros Briasoulis

**Affiliations:** 1 Department of Cardiology, University Hospital of Larissa, Faculty of Medicine, University of Thessaly, Larissa, Greece; 2 School of Medicine, European University Cyprus, Nicosia, Cyprus; 3 Department of Cardiology, 417 Army Share Fund Hospital of Athens (NIMTS), Athens, Greece; 4 Department of Cardiac Surgery Research, Lankenau Institute for Medical Research, Wynnewood, PA, United States; 5 Department of Clinical Therapeutics, National and Kapodistrian University of Athens, Athens, Greece

**Keywords:** heart failure, heart transplantation, left ventricular assist device, pulmonary hypertension, pulmonary vascular resistance, right ventricular failure, sotatercept

## Introduction: from a PAH drug to a pulmonary vascular strategy

Sotatercept represents a mechanistic departure from conventional pulmonary arterial hypertension (PAH) therapies. Rather than augmenting nitric oxide, endothelin, or prostacyclin pathways to produce vasodilation, it acts as a ligand trap for selected activin and growth differentiation factor ligands, thereby rebalancing pro-proliferative and anti-proliferative signaling within the transforming growth factor-β superfamily (1–3). In PAH, this approach improves exercise capacity, functional class, pulmonary hemodynamics, and clinical outcomes when added to background therapy (1, 2, 4, 5). Its significance may nevertheless extend beyond World Health Organization group 1 disease.

Pulmonary vascular remodeling and right ventricular (RV) maladaptation are shared determinants of outcome across several advanced heart failure states. They occur in a subset of patients with heart failure with preserved ejection fraction (HFpEF), in advanced heart failure with reduced ejection fraction (HFrEF), after left ventricular assist device (LVAD) implantation, and before or after heart transplantation. The central question is therefore not whether sotatercept should become a treatment for “heart failure” broadly. It is whether a pulmonary vascular phenotype, persistent elevation of pulmonary vascular resistance (PVR), impaired RV-pulmonary arterial (PA) coupling, and RV dysfunction, despite appropriate control of left-sided filling pressures, may identify patients in whom activin-pathway modulation deserves investigation.

## Lessons from PAH: remodeling, coupling, and the right ventricle

Evidence from PAH provides both the biological rationale and the clinical foundation for this hypothesis. In the phase 2 PULSAR trial, sotatercept, added to established PAH therapy, reduced pulmonary vascular resistance (PVR) and improved six-minute walk distance (1). These findings were reinforced by STELLAR, which demonstrated a clinically meaningful improvement in exercise capacity, together with broad secondary benefits, including reductions in PVR and N-terminal pro-B-type natriuretic peptide, improvement in functional class, and a lower risk of clinical worsening (2). In ZENITH, sotatercept reduced the composite of death, lung transplantation, or hospitalization for worsening PAH in high-risk patients (17.4% vs. 54.7%; hazard ratio, 0.24) (6). Similarly, in HYPERION, sotatercept reduced clinical worsening within the first year after diagnosis (10.6% vs. 36.9%; hazard ratio, 0.24) (7). Consistent with these findings, a meta-analysis of four randomized trials (*n* = 889) reported a 77% lower risk of clinical worsening or death and favorable functional and hemodynamic effects (8). Importantly, a detailed analysis of STELLAR indicated that the reduction in PVR was driven predominantly by a fall in pulmonary artery pressure and was accompanied by reverse right-heart remodeling and improved RV function (4). These findings establish hemodynamic unloading; they do not, by themselves, prove structural pulmonary vascular reverse remodeling.

Emerging observations suggest that the response may begin earlier than structural reverse remodeling alone would predict. Continuous invasive pressure monitoring in five sotatercept-treated patients showed a rapid decline in RV pressure and improvement in RV-PA interaction after the first dose, although the sample was extremely small and nonrandomized (9). In a separate cardiovascular magnetic resonance cohort of 18 intermediate-high-risk patients, RV end-diastolic and end-systolic volumes decreased by 34 and 27 mL, respectively, while RV ejection fraction increased by five percentage points after approximately 24 weeks (10). These imaging observations suggest possible reverse remodeling but require confirmation in larger controlled studies. Taken together, these findings position the RV–PA unit as the most relevant bridge to advanced heart failure and transplantation.

## CADENCE: proof of concept in HFpEF-associated CpcPH

The strongest evidence beyond PAH comes from CADENCE, the phase 2 randomized trial in combined post- and precapillary pulmonary hypertension associated with HFpEF (CpcPH-HFpEF) (11). Among 164 participants with a median baseline PVR of 5.2 Wood units, sotatercept reduced PVR at 24 weeks compared with placebo. The placebo-adjusted Hodges–Lehmann estimates were −1.02 Wood units with 0.3 mg/kg and −0.75 Wood units with 0.7 mg/kg. Both doses reduced mean pulmonary artery pressure by approximately 9 mmHg. The 0.3-mg/kg dose also reduced pulmonary artery wedge pressure (PAWP) by approximately 3 mmHg and increased six-minute walk distance by 20.3 m relative to placebo.

CADENCE is important for three reasons. First, it establishes that activin-signaling inhibition can modify hemodynamics in carefully selected group 2 pulmonary hypertension, a setting in which PAH vasodilators have frequently been neutral or harmful. Second, PVR fell despite a reduction in resting cardiac output, while PAWP, right atrial pressure, left atrial volume, N-terminal pro-B-type natriuretic peptide, pulmonary arterial compliance, and indices of RV-PA coupling generally moved favorably (11). Third, the lower dose appeared at least as effective as the higher dose, emphasizing that more intense ligand trapping may not necessarily produce greater clinical benefit.

Packer has interpreted these findings through the “adipokine hypothesis” of HFpEF: activin A derived from dysfunctional epicardial adipose tissue may promote both myocardial fibrosis and pulmonary vascular disease, providing a potential explanation for simultaneous improvement in PVR, left atrial volume, and filling pressure (12). This is biologically attractive, particularly in an obese HFpEF population, but remains an interpretation rather than a demonstrated human mechanism. CADENCE should therefore not be generalized to all HFpEF. Its message is narrower and more powerful: hemodynamically confirmed CpcPH may define a treatable pulmonary vascular phenotype within HFpEF.

## From HFpEF to advanced HFrEF

To date no clinical study directly evaluates sotatercept in HFrEF. Extrapolation must therefore remain cautious. In advanced HFrEF, pulmonary hypertension initially reflects elevated left atrial and pulmonary venous pressure. With sustained exposure, endothelial dysfunction, inflammation, fibrosis, and arterial as well as venous remodeling may produce a precapillary component and rising PVR (13). This transition carries prognostic importance because the RV faces both increased afterload and reduced coronary perfusion reserve.

Sotatercept should not be considered while congestion and high PAWP remain inadequately treated. Increasing transpulmonary flow across an unprepared left ventricle could worsen pulmonary venous pressure, as has occurred with pulmonary-selective vasodilators in left-heart disease (14). The rational population would instead comprise patients receiving optimized guideline-directed therapy who have reproducible CpcPH, disproportionate PVR elevation, and RV-PA uncoupling after decongestion. In this setting, the goal would not be conventional HFrEF treatment, but modification of a persistent pulmonary vascular barrier to recovery, mechanical support, or transplantation. Although pooled PULSAR and STELLAR data indicated consistent efficacy and safety across cardiac-index strata in PAH, these findings cannot be extrapolated to advanced HFrEF; any use in this population should currently be confined to appropriately designed clinical studies (15).

## LVAD support

LVAD implantation unloads the left ventricle and often reduces PAWP, pulmonary artery pressure, and PVR. Yet pulmonary vascular disease does not regress uniformly. Some patients retain elevated PVR despite adequate unloading, while others develop early or late RV failure because increased venous return, septal shift, intrinsic RV dysfunction, and residual pulmonary afterload exceed RV reserve (13). These patients offer a particularly informative setting in which left-sided congestion can be separated, albeit imperfectly, from persistent pulmonary vascular remodeling.

Experience with phosphodiesterase-5 inhibitors (PDE5i) supports the general premise that postimplant pharmacological modulation of the pulmonary circulation may influence LVAD outcomes, but it cannot establish a sotatercept effect. In an INTERMACS analysis of 13,772 continuous-flow LVAD recipients, postimplant PDE5i use was associated with fewer thrombotic events and lower mortality, at the cost of more gastrointestinal bleeding (16). In 7,229 recipients of centrifugal-flow devices, PDE5i use was associated with lower mortality and ischemic stroke, although pump thrombosis was unchanged (17). These observational associations require randomized confirmation and should not be interpreted as a class effect (18, 19).

The testable sotatercept hypothesis is more specific: after LVAD speed, volume status, valvular disease, arrhythmias, and conventional RV-directed therapy have been optimized, could sotatercept reduce persistent PVR, improve RV-PA coupling, prevent late RV failure, or convert a patient from transplant ineligibility to candidacy? An early study should enroll only patients with invasive confirmation of adequate LV unloading and residual precapillary disease. Serial right-heart catheterization, echocardiographic or pressure-derived RV-PA coupling, hemoglobin, platelet count, bleeding, thrombosis, pump parameters, and functional capacity should be prespecified. Given the small LVAD population and event heterogeneity, a multicenter adaptive or registry-based randomized design may be more feasible than a conventional large trial (18).

## Transplant candidacy and post-transplant RV failure

Elevated, insufficiently reversible PVR remains a major barrier to heart transplantation because an unconditioned donor RV may fail when exposed to high afterload. LVAD support can restore candidacy by unloading the left heart, but persistent PVR may require additional pulmonary vascular therapy. Sotatercept could therefore be investigated as a bridge-to-candidacy strategy following adequate unloading, once persistent pulmonary hypertension has been shown to reflect predominantly residual pulmonary vascular disease.

The post-transplant setting is even more speculative but clinically compelling. A 2026 conference case described a 43-year-old woman who developed severe RV failure after heart transplantation, with recurrent PVR of 5 Wood units, vasopressor and inhaled nitric oxide dependence, and limited response to sildenafil because of systemic hypotension (20). After sotatercept 0.3 mg/kg, vasopressors and inhaled therapy were discontinued within one week, RV function improved, and the patient was discharged. This single, uncontrolled case cannot establish efficacy; concomitant therapies and natural postoperative recovery are major confounders. Nevertheless, it illustrates why a nonconventional, potentially less systemically vasodilating strategy could be attractive when elevated PVR drives post-transplant RV failure.

Future investigation should proceed sequentially: first in stable LVAD-supported candidates with persistent PVR, then in carefully monitored peri-transplant cohorts, and only later in rescue RV failure. Interaction with immunosuppression, infection, renal dysfunction, wound healing, allograft biology, and rejection remains unknown.

## A staged translational agenda

The breadth of the hypothesis should not lead to a single heterogeneous “all-comers” trial. A staged program could answer distinct questions while protecting patients. The first step should be a mechanistic study in clinically stable patients with advanced HF, preferably including an LVAD-supported stratum, in whom repeated invasive assessment confirms PAWP control but persistent PVR elevation. Proposed endpoints could include changes in PVR, pulmonary arterial compliance, RV stroke work, cardiac output reserve, and an objective index of RV–PA coupling. Exercise hemodynamics may be particularly informative, given that resting cardiac output decreased in CADENCE despite improved exercise capacity (11). Biomarkers and imaging may explore whether changes arise from pulmonary vascular remodeling, altered plasma volume, higher hemoglobin, myocardial effects, or a combination of mechanisms.

If initial studies suggest hemodynamic benefit and acceptable short-term safety, a bridge-to-candidacy trial could then be considered. Such a study might include blinded transplant-committee assessment of whether elevated PVR had previously limited eligibility and whether treatment achieved prespecified hemodynamic criteria. Changes in PVR should be interpreted alongside PAWP, systemic blood pressure, forward flow, and hematologic safety. The durability of benefit after treatment interruption and post-transplant donor RV function could provide additional clinically relevant information.

Post-transplant evaluation could initially be observational or conducted within carefully governed compassionate-use protocols. Patient selection should distinguish pulmonary vascular afterload from other causes of early graft dysfunction, including primary graft dysfunction, rejection, tamponade, ischemia, volume overload, and intrinsic donor-heart dysfunction. If a randomized rescue trial were later considered, early feasibility endpoints might include liberation from vasoactive and inhaled pulmonary vasodilator support, changes in PVR and RV function, renal recovery, and serious adverse events, as survival or retransplantation would likely require substantially larger studies. Independent event adjudication and standardized definitions of mechanical circulatory support would strengthen evaluation across all stages (18).

## Safety may differ across the continuum

The safety profile observed in PAH cannot simply be transferred to advanced heart failure, LVAD recipients, or transplant recipients. In a meta-analysis of four randomized PAH trials, total bleeding occurred in 37.9% with sotatercept versus 18.7% with placebo, epistaxis in 26.7% versus 5.4%, telangiectasia in 19.8% versus 6.4%, increased hemoglobin in 10.0% versus 0.7%, and thrombocytopenia in 8.9% versus 4.0% (8). Increased hemoglobin, thrombocytopenia, epistaxis and other bleeding, telangiectasia, hypertension, and dose interruptions are established concerns (1, 2, 10, 11). The CMR cohort reported new or enlarging pericardial effusion in 5 of 18 patients, without hemodynamic compromise; this association requires confirmation but could be diagnostically challenging after LVAD implantation or transplantation (10).

In LVAD recipients, competing hazards are especially important. Erythrocytosis could theoretically enhance oxygen delivery but may also increase blood viscosity; thrombocytopenia and vascular lesions could amplify bleeding. No current evidence defines the net hemocompatibility effect of sotatercept during continuous-flow support. Similarly, transplant recipients may have cytopenias, renal impairment, infection, or concomitant antithrombotic therapy that alters tolerability. Safety endpoints must therefore be co-primary rather than secondary considerations in early trials.

## Conclusion

Sotatercept should not yet be viewed as a treatment for the full spectrum of heart failure. A more precise proposition is that activin-pathway modulation may target pulmonary vascular remodeling and RV-PA uncoupling wherever these abnormalities persist despite correction of left-sided loading conditions (Figure 1). CADENCE provides randomized proof of concept in CpcPH-HFpEF. PAH trials establish favorable pulmonary hemodynamic effects across a broad spectrum of disease severity, while controlled evidence of structural reverse remodeling remains limited. LVAD and transplant applications remain hypotheses supported by pathophysiology and isolated clinical signals. The transplant continuum offers a coherent program for testing this proposition, from selected CpcPH, through LVAD-supported persistent PVR and transplant candidacy, to post-transplant RV failure. The next step is not therapeutic extrapolation, but disciplined, phenotype-enriched trials.

**Figure 1:**
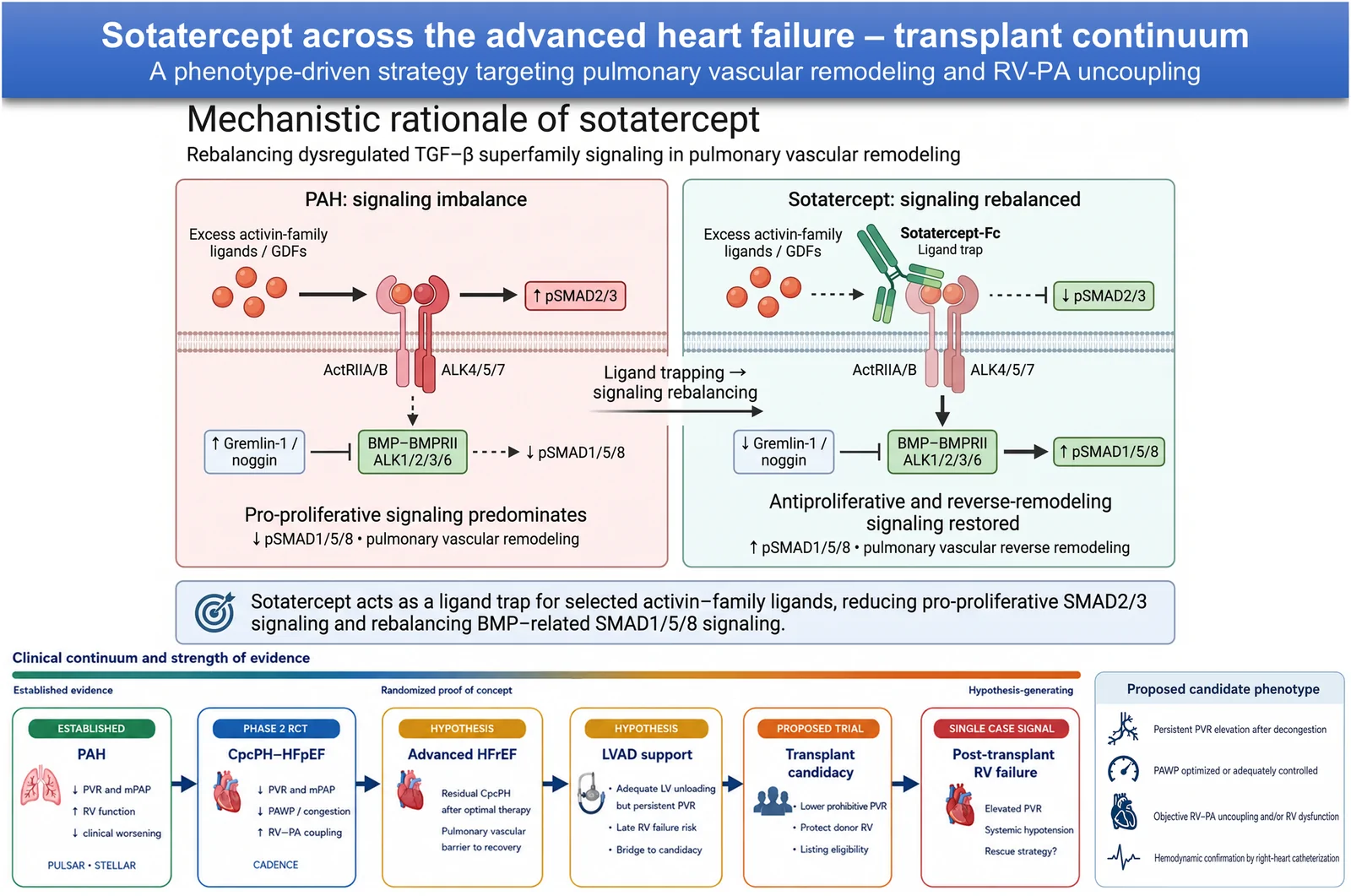
Sotatercept across the advanced heart failure and transplant continuum. The proposed therapeutic continuum begins with established evidence in PAH and randomized proof of concept in CpcPH-HFpEF, then progresses through increasingly hypothesis-generating settings: advanced HFrEF with persistent pulmonary vascular remodeling, residual CpcPH during LVAD support, restoration of transplant candidacy, and post-transplant RV failure. Across stages, the proposed treatment target is persistent elevation of PVR with RV–PA uncoupling after optimization of left-sided filling pressures. The evidence gradient is displayed explicitly. CpcPH, combined post- and precapillary pulmonary hypertension; HFpEF, heart failure with preserved ejection fraction; HFrEF, heart failure with reduced ejection fraction; LVAD, left ventricular assist device; PAH, pulmonary arterial hypertension; PAWP, pulmonary artery wedge pressure; PVR, pulmonary vascular resistance; RV–PA, right ventricular–pulmonary arterial.

## References

[1] Humbert M McLaughlin V Gibbs JSR Gomberg-Maitland M Hoeper MM Preston IR. Sotatercept for the treatment of pulmonary arterial hypertension. N Engl J Med. (2021) 384(13):1204–15. 10.1056/NEJMoa2024277 33789009

[2] Hoeper MM Badesch DB Ghofrani HA Gibbs JSR Gomberg-Maitland M McLaughlin VV. Phase 3 trial of sotatercept for treatment of pulmonary arterial hypertension. N Engl J Med. (2023) 388(16):1478–90. 10.1056/NEJMoa2213558 36877098

[3] Truong R Davis S Rosario N Wollen J Johnson C Wang Y. Sotatercept: a new frontier in pulmonary arterial hypertension treatment. Ann Pharmacother. (2026) 60(2):163–74. 10.1177/10600280251358959 40876858

[4] Souza R Badesch DB Ghofrani HA Gibbs JSR Gomberg-Maitland M McLaughlin VV. Effects of sotatercept on haemodynamics and right heart function: analysis of the STELLAR trial. Eur Respir J. (2023) 62(3):2301107. 10.1183/13993003.01107-2023 37696565 PMC10512088

[5] Filippatos C Boutsikos I Tepetes N-I Xanthopoulos A Duque ER Asleh R. Sotatercept as an add-on to background therapy in idiopathic pulmonary arterial hypertension: insights from a real-world cohort. Pharmaceuticals (Basel). (2026) 19(5):760. 10.3390/ph19050760 42198434 PMC13209685

[6] Humbert M McLaughlin VV Badesch DB Ghofrani HA Gibbs JSR Gomberg-Maitland M. Sotatercept in patients with pulmonary arterial hypertension at high risk for death. N Engl J Med. (2025) 392(20):1987–2000. 10.1056/NEJMoa2415160 40167274

[7] McLaughlin VV Hoeper MM Badesch DB Ghofrani HA Gibbs JSR Gomberg-Maitland M. Sotatercept for pulmonary arterial hypertension within the first year after diagnosis. N Engl J Med. (2025) 393(16):1599–611. 10.1056/NEJMoa2508170 41025556

[8] Murad MR Awashra A Elgendy MS Kasem AW Shubietah A Safi F. Clinical, hemodynamic, and safety outcomes of sotatercept in pulmonary arterial hypertension: a meta-analysis of randomized trials with time-to-event data. Ther Adv Respir Dis. (2026) 20:17534666261459209. 10.1177/17534666261459209 42312805 PMC13287412

[9] Kremer N Thal BR Janetzko P Rako ZA Yogeswaran A Bonnet S. Acute hemodynamic effects of sotatercept. Circulation. (2025) 152(24):1735–8. 10.1161/CIRCULATIONAHA.125.076913 41205220

[10] Virsinskaite R Beattie CJ Karia N Kotecha T Schreiber BE Suntharalingam J. Sotatercept improves right ventricular function but is associated with new or worsening pericardial effusions: a cardiovascular magnetic resonance study in intermediate-high risk pulmonary arterial hypertension. J Cardiovasc Magn Reson. (2026) 28(1):102714. 10.1016/j.jocmr.2026.102714 41864345 PMC13241712

[11] Gomberg-Maitland M Tedford RJ Langleben D Rosenkranz S Miller B Jones AD. Sotatercept for combined post- and precapillary pulmonary hypertension associated with heart failure: results from the phase 2, randomized, placebo-controlled CADENCE study. Circulation. (2026) 153(19):1446–59. 10.1161/CIRCULATIONAHA.126.079918 41904795

[12] Packer M. The CADENCE trial: activin type II receptor ligand trapping with sotatercept as first prospective proof-of-concept for the adipokine hypothesis of heart failure with preserved ejection fraction. J Card Fail. (2026). 10.1016/j.cardfail.2026.04.010. [Online ahead of print]. 42067121

[13] Triposkiadis F Xanthopoulos A Skoularigis J Starling RC. Therapeutic augmentation of NO-sGC-cGMP signalling: lessons learned from pulmonary arterial hypertension and heart failure. Heart Fail Rev. (2022) 27(6):1991–2003. 10.1007/s10741-022-10239-5 35437713

[14] Kiyohara Y Sakai T Freeman LM Katzianer D Siuba MT Chaisson N. Pulmonary arterial hypertension-targeted therapy for pulmonary hypertension associated with left heart disease: Bayesian and frequentist meta-analysis of randomized controlled trials. Am J Cardiol. (2026). 10.1016/j.amjcard.2026.07.022. [Online ahead of print]. 42551818

[15] Gomberg-Maitland M Badesch DB Gibbs JSR Grünig E Hoeper MM Humbert M. Efficacy and safety of sotatercept across ranges of cardiac index in patients with pulmonary arterial hypertension: a pooled analysis of PULSAR and STELLAR. J Heart Lung Transplant. (2025) 44(4):609–24. 10.1016/j.healun.2024.11.037 39645016

[16] Xanthopoulos A Tryposkiadis K Triposkiadis F Fukamachi K Soltesz EG Young JB. Postimplant phosphodiesterase type 5 inhibitors use is associated with lower rates of thrombotic events after left ventricular assist device implantation. J Am Heart Assoc. (2020) 9(14):e015897. 10.1161/JAHA.119.015897 32648508 PMC7660717

[17] Xanthopoulos A Wolski K Wang Q Blackstone EH Randhawa VK Soltesz EG. Postimplant phosphodiesterase-5 inhibitor use in centrifugal flow left ventricular assist devices. JACC Heart Fail. (2022) 10(2):89–100. 10.1016/j.jchf.2021.09.008 35115092

[18] Xanthopoulos A Fukamachi K Starling RC. Challenges for research in the LVAD population. JACC Heart Fail. (2025) 13(10):102604. 10.1016/j.jchf.2025.102604 40811936

[19] Xanthopoulos A Magouliotis DE Triposkiadis F Starling RC. From small cavities to big gains: phosphodiesterase 5 inhibitors in LVAD care. JACC Heart Fail. (2026) 14(1):102756. 10.1016/j.jchf.2025.102756 41500729

[20] Fabbri M Celenza-Salvatore J Contreras J Moss N Youssef J Correa A. Sotatercept for right ventricular failure following heart transplantation in a patient with elevated pulmonary vascular resistance: a case report (conference abstract). J Heart Lung Transplant. (2026) 45:377. 10.1016/j.healun.2026.02.819

